# Hypoxia-Induced Mesenchymal Stem Cells Exhibit Stronger Tenogenic Differentiation Capacities and Promote Patellar Tendon Repair in Rabbits

**DOI:** 10.1155/2020/8822609

**Published:** 2020-10-17

**Authors:** Guanyin Chen, Wangqian Zhang, Kuo Zhang, Shuning Wang, Yuan Gao, Jintao Gu, Lei He, Weina Li, Cun Zhang, Wei Zhang, Meng Li, Qiang Hao, Yingqi Zhang

**Affiliations:** State Key Laboratory of Cancer Biology, Biotechnology Center, School of Pharmacy, Fourth Military Medical University, No. 169, Changle West Road, Xi'an 710032, China

## Abstract

Tendon injury is a common but tough medical problem. Unsatisfactory clinical results have been reported in tendon repair using mesenchymal stem cell (MSC) therapy, creating a need for a better strategy to induce MSCs to tenogenic differentiation. This study was designed to examine the effect of hypoxia on the tenogenic differentiation of different MSCs and their tenogenic differentiation capacities under hypoxia condition in vitro and to investigate the in vivo inductility of hypoxia in tenogenesis. Adipose tissue-derived MSCs (AMSCs) and bone marrow-derived MSCs (BMSCs) were isolated and characterized. The expression of hypoxia-induced factor-1 alpha (Hif-1*α*) was examined to confirm the establishment of hypoxia condition. qRT-PCR, western blot, and immunofluorescence staining were used to evaluate the expression of tendon-associated marker Col-1a1, Col-3a1, Dcn, and Tnmd in AMSCs and BMSCs under hypoxia condition, compared with Tgf-*β*1 induction. In vivo, a patellar tendon injury model was established. Normoxic and hypoxic BMSCs were cultured and implanted. Histological, biomechanical, and transmission electron microscopy analyses were performed to assess the improved healing effect of hypoxic BMSCs on tendon injury. Our in vitro results showed that hypoxia remarkably increased the expression of Hif-1*α* and that hypoxia not only promoted a significant increase in tenogenic markers in both AMSCs and BMSCs compared with the normoxia group but also showed higher inductility compared with Tgf-*β*1. In addition, hypoxic BMSCs exhibited higher potential of tenogenic differentiation than hypoxic AMSCs. Our in vivo results demonstrated that hypoxic BMSCs possessed better histological and biomechanical properties than normoxic BMSCs, as evidenced by histological scores, patellar tendon biomechanical parameters, and the range and average of collagen fibril diameters. These findings suggested that hypoxia may be a practical and reliable strategy to induce tenogenic differentiation of BMSCs for tendon repair and could enhance the effectiveness of MSCs therapy in treating tendon injury.

## 1. Introduction

Tendons are special connective tissues that transmit the force from muscles to bones, playing a key role in the musculoskeletal system. Tendon injury is a very common soft tissue injury, afflicting about 30 million people each year and accounting for up to 50% of all sport-related injuries [1, 2]. Not only is tendon injury prevalent but also is it a tough clinic problem, due to its hard healing process because of hypovascularity and hypocellularity in the tendon [3]. Many attempts, such as autologous grafting, application of growth factors, and gene therapy, have been made to repair the injury, but often following the dysfunction of the donor site and a long and poor recovery [4, 5]. Therefore, the treatment strategy to repair tendon injury still needed to be explored.

Mesenchymal stem cell (MSC) therapy has shown promise in treating tendon injury [6]. However, the findings about the effect of undifferentiated MSCs on repairing tendon are inconsistent [7, 8]. No significant differences were found between the untreated group and the adipose tissue-derived mesenchymal stem cell (AMSC) group in all biomechanical variables at 2 and 4 weeks after surgery in a rotator cuff injury model [7]. Another study found that there was only a significant increase in the maximum load and stiffness in the bone marrow-derived mesenchymal stem cell (BMSC) group compared with the control group at 2 weeks after surgery [8]. By contrast, other studies found that application of AMSCs or BMSCs could improve fiber arrangement and enhance tensile strength at all timepoints postoperatively [9, 10]. Tenogenically differentiated MSCs could improve tendon repair in both histological scores and biomechanical properties [6]. Many findings have shown that transforming growth factor-*β*1 (Tgf-*β*1) can promote tenogenic differentiation of MSCs and thus improve tendon repair [11, 12]. BMSCs transfected with the Tgf-*β*1 gene showed higher concentrations of collagen type I protein, larger fiber bundles, and more rapid matrix remodeling [11]. Recent study found that tenocyte induced tenogenic differentiation of BMSCs by secreting exosomes containing Tgf-*β*1 [12]. BMP-induced tenogenic differentiation of AMSCs enhanced the expression of collagen type I and scleraxis and exhibited improved histological score and collagen fiber dispersion range compared to undifferentiated AMSCs [13]. Although the induction capacities were low, insulin-like growth factor 1 and basic fibroblast growth factor could still promote BMSCs to differentiate into tenocytes by upregulating collagen type I/III and scleraxis [14, 15]. However, healed tendon rarely achieves complete functionality equal to the preinjured state. The final biomechanical properties of repaired tendon may be reduced by more than 30% because of the low regenerative capacity of tenocytes [16] and the complicated tenogenic differentiation process of MSCs [6], which make it difficult to promote tendon repair by improving the existing methods. As a result, a study on looking for new strategy to induce tenogenic differentiation is imperative.

Hypoxia is the microenvironment of both tenocytes and MSCs. The oxygen tension has been shown to be 1-10% in the tendon [17], 1–2% in the bone marrow, and approximately 3% in adipose tissue [18]. Previous findings have shown that hypoxia could promote the stemness of MSCs [19]. However, whether hypoxic MSCs could increase their differentiation potential to adipocyte, osteocyte, and chondrocyte in vitro and in vivo has been controversial [17, 20]. As for tenogenic differentiation, only few evidences showed that the mRNA levels of tenomodulin and tenascin-C were increased under hypoxia condition [17, 21]. In addition, normoxic BMSC resulted in better ultrasound echogenicity score and upregulation of collagen type I, decorin, and tenascin-C mRNAs than normoxic AMSC in vivo [22, 23]. However, whether hypoxia could induce MSCs to differentiate into tenocytes and the difference of tenogenic differentiation capacities between different MSCs in hypoxia induction have not been systematically investigated, which limits the knowledge for treatment of tendon injury.

The purpose of this study was to investigate the role of hypoxia in tenogenic differentiation of AMSCs and BMSCs compared with Tgf-*β*1, an effective inducer of tenogenesis [24], and the difference of tenogenic differentiation capacities between AMSCs and BMSCs under hypoxia condition in vitro and to compare the effect of normoxic BMSCs and hypoxic BMSCs on tenogenesis in vivo. Our results suggested that hypoxia could promote tenogenesis of AMSCs and BMSCs more effectively than Tgf-*β*1 and that hypoxic BMSCs showed higher tenogenic differentiation capacities than hypoxic AMSCs. Our results also showed that hypoxic BMSCs exhibited better histological and biomechanical properties than normoxic BMSCs.

## 2. Materials and Methods

### 2.1. Animals

**Figure 1:**
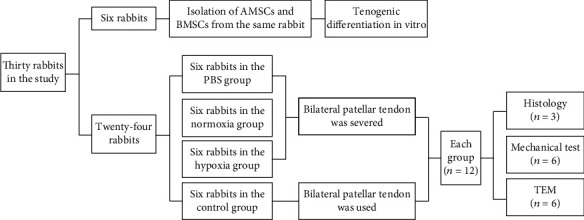
Schematic flow chart of experimental design and groups of the study.

A total of thirty New Zealand rabbits were used in this experiment (2.5–3.5 kg, 3–5 months old). Six rabbits were used for isolating AMSCs and BMSCs. AMSCs and BMSCs from the same rabbit were tested in this study. The rest twenty-four rabbits were randomly divided into the phosphate buffer saline (PBS) group, the normoxia group, the hypoxia group, and the control (normal) group. A flow chart showing the grouping of animals and the sample size for each experiment was shown in Figure 1. Before the experiment, each rabbit was checked for general health. During the following experiment, each rabbit was housed in a commercial animal cage (49 cm × 35 cm × 32 cm) with 12 h/12 h light/dark cycle, kept at room temperature with free access to food and water. All animal experiments were approved by the Ethics Committees of the Fourth Military Medical University and were conducted according to the Guideline of Animal Care and Use Committee of the Fourth Military Medical University. Every effort was made to minimize the use and the discomfort of the rabbits.

### 2.2. Isolation and Culture of AMSCs and BMSCs

AMSCs were isolated from the inguinal adipose tissue digested by DME/F12 medium containing 0.2% collagenase type I (Sigma, USA) in 37°C for 2 h. The tissue residues were filtered by 200-mesh sieve, centrifuged at 350 x g for 5 min, resuspended in DME/F12 complete medium containing 15% fetal bovine serum (FBS, Gibco, USA) and 1% penicillin/streptomycin/amphotericin B (Cellmaxin plus, Gendepot, USA), and plated onto 10 cm cell culture dishes at 37°C with 5% CO_2_. The medium was changed every 2 days, and the cells (passage 0, P0) were subcultured when 90% confluence was reached. BMSCs were collected from the bone marrow extruded by inserting a 22-gauge needle into the shaft of the femora and flushed out with DME/F12 medium containing 15% FBS and 1% penicillin/streptomycin/amphotericin B. After repetitively pipetting, the medium containing the whole bone marrow was then plated onto 10 cm cell culture dishes at 37°C with 5% CO_2_. After 48 h, half of the initial culture was changed by fresh culture medium. The cells (P0) typically reached 80-90% confluence within 9 days and were harvested for subcultures. The P3 cells were used in the experiment.

### 2.3. Flow Cytometry

Flow cytometry analysis was taken to confirm surface antigen marker of MSCs. 1 × 10^6^ cells at P3 in the logarithm growth period were collected, washed twice with 1% pre-cooled FBS/PBS, and centrifuged at 350 x g for 5 min. Cells were incubated with anti-CD34-PE (GeneTex, USA), anti-CD45-APC (Invitrogen, USA), anti-CD44-APC (Novus Biologicals, USA), and anti-CD29-FITC (Invitrogen, USA) for 30 min at 4°C in the dark, respectively. Labeled cells were washed twice and examined using the FACScan flow cytometry system (BD, Franklin Lakes, USA). Data were analyzed with the FlowJo software (TreeStar, Ashland, OR, USA). PBS solution was used for negative control staining.

### 2.4. Immunofluorescence Staining

MSCs were cultured on laser confocal dishes, fixed with 4% formalin for 15 min when they reached 60% confluence, blocked with Immunol Staining Blocking Buffer (containing Triton X-100 for permeabilization; Beyotime, China) for 1 h, and followed by incubating with hypoxia induced factor-1 alpha (Hif-1*α*, 1 : 300, Bioss, Beijing, China) and tenomodulin (Tnmd, 1 : 400, Bioss, Beijing, China) primary antibody solution overnight at 4°C. After washing 3 times with PBS, the cells were incubated with Cy3-goat anti-rabbit IgG (Beijing ComWin Biotech Co., Ltd., China) at room temperature for 1 h in dark place, followed by counterstaining with DAPI for 5 min. The prepared samples were observed under laser scanning confocal microscope (Nikon A1R, Japan). The images (each sample for at least 3 fields) were analyzed with ImagePro Plus version 6.0 (Media Cybernetics, Inc.). The average optical density (AOD) was equal to integrated optical density over area. For CD molecular identification, the cells were blocked with normal goat serum prior to incubation with the following antibodies: CD34-PE (GeneTex, USA), CD45-APC (Invitrogen, USA), CD29-FITC (Invitrogen, USA), and CD90-FITC (BioLegend, USA) overnight at 4°C in dark place.

### 2.5. Chondrogenic, Adipogenic, and Osteogenic Differentiation

For chondrogenic differentiation, 3 × 10^5^ MSCs were collected and washed 3 times with chondrogenic induction medium (percent by volume) consisting of 0.01% dexamethasone, 0.3% ascorbate, 1% ITS + supplement, 0.1% sodium pyruvate, 0.1% proline, and 1% Tgf-*β*3 in chondrogenic basal medium (RBXMX-90042, Cyagen, China) under centrifugation at 350 x g for 5 min. The pellet was cultured in 0.5 ml chondrogenic induction medium in the 15-ml tube. After 24 h, the pellet was suspended by slightly knocking the bottom of the tube. The medium was replaced every 2 days, and the pellet was cultured for 28 days. For the staining, the pellet was fixed with 4% formalin, embedded, dewaxed, dehydrated, and stained with Alcian Blue 8GX solution for 30 min. For adipogenic differentiation, the cells were seeded at a density of 2 × 10^4^ cells/cm^2^ and cultured in DME/F12 complete medium until they reached 100% confluence. Then, the culture medium was changed with adipogenic induction medium A (percent by volume) consisting of 1% glutamine, 0.2% recombinant human insulin, 0.1% 3-isobutyl-1-methyl-xanthine, 0.1% rosiglitazone, 0.1% dexamethasone, and 10% FBS in adipogenic basal medium (RBXMX-90031, Cyagen, China). After 72 h, the medium was changed with adipogenic induction medium B (percent by volume) consisting of 1% glutamine, 0.2% recombinant human insulin, and 10% FBS in adipogenic basal medium (RBXMX-90031, Cyagen, China). After 24 h, the medium was changed with adipogenic induction medium A. After repeating 5 times, the cells were cultured with adipogenic induction medium B for 7 days. For the staining, the cells were fixed with 4% formalin for 30 min, washed, and stained with Oil Red O solution for 30 min. For osteogenic differentiation, the bottom of the culture dish was covered by 0.1% gelatin in order to prevent cell shedding. MSCs were seeded at a density of 2 × 10^4^ cells/cm^2^ and cultured in DME/F12 complete medium until they reached 60%-70% confluence. Then, the culture medium was changed with osteogenic induction medium (percent by volume) including 1% glutamine, 0.1% dexamethasone, 1% *β*-glycerophosphate, 0.2% ascorbate, and 10% FBS in osteogenic basal medium (RBXMX-90021, Cyagen, China). The medium was replaced every 2 days during the induction process which lasted for 28 days. For the staining, the cells was fixed with 4% formalin for 30 min, washed, and stained with Alizarin Red solution for 5 min.

### 2.6. Tenogenic Inductive Protocol

Under hypoxia condition, cells were cultured in complete medium in a trigas incubator maintained at 1% O_2_, 5% CO_2_, and 94% N_2_ (MCO-5 M, SANYO, Japan) for 7 consecutive days. The medium was changed every 2 days. Whereas in Tgf-*β*1 condition, cells were cultured in complete medium containing 10 ng/ml Tgf-*β*1 (Sigma, USA) for 7 consecutive days. The medium containing Tgf-*β*1 was also changed every 2 days.

### 2.7. Quantitative Real-Time Polymerase Chain Reaction (qRT-PCR)

The mRNA levels of tenogenic genes were measured using quantitative real-time PCR (qRT-PCR). Total RNA was extracted using RNAiso plus according to the manufacturer's protocol (TaKaRa, Japan). cDNA was synthesized using a reverse transcription kit (TaKaRa, Japan). qRT-PCR analysis was performed using the CFX96 Real-Time PCR Detection System (Rotor-Gene Q 2plex, Germany). The conditions of qRT-PCR were as follows: 95°C for 30 s, followed by 39 cycles of 95°C for 5 s and 60°C for 30 s, and a final extension at 95°C for 10 s. The primers were synthesized by Sangon Biotech Co., Ltd. (Shanghai, China), and the sequences of the primers were shown in Table 1. No nonspecific amplification was found by the melting curve. Data were acquired from at least five independent samples and tested at least three times. The mRNA expression levels relative to *β*-actin were quantified using the 2^−*ΔΔ*CT^ method.

### 2.8. Western Blot

Proteins were extracted using RIPA Lysis Buffer (Shanghai Weiao Biotechnology Co., Ltd., China), and the concentration was measured using the BCA protein reagent kit (Beijing Solarbio Science & Technology Co., Ltd., China). Proteins (30 *μ*g) were separated on SDS-PAGE gels (8%), transferred onto a polyvinylidene difluoride (PVDF) membrane, and blocked with 5% skim milk for 1 h at room temperature. The PVDF membranes were incubated with anti-Hif-1*α* (1 : 400), anti-Collagen type I (Col-1a1, 1 : 400), anti-Collagen type III (Col-3a1, 1 : 400), anti-Decorin (Dcn, 1 : 400), and anti-Tnmd (1 : 400) primary antibody solution (Bioss, Beijing, China) and anti-*β*-Actin (1 : 1000) primary antibody solution (BOSTER Biological Technology Co., Ltd., China) overnight at 4°C. After incubating with horseradish peroxidase-conjugated goat anti-rabbit secondary antibody or goat anti-mouse secondary antibody (BOSTER Biological Technology Co., Ltd., China) at room temperature for 1 h, the membranes were reacted with ECL hypersensitive chemiluminescence kit (Shanghai Weiao Biotechnology Co., Ltd., China) according to the manufacturer's protocol.

### 2.9. Tendon Injury Model

**Figure 2:**
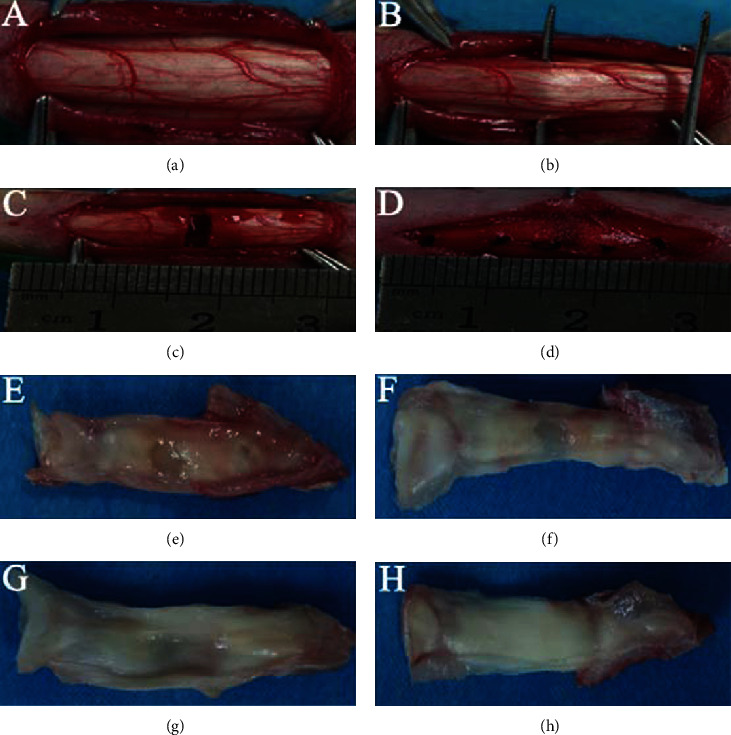
Surgical procedure for the patellar tendon injury model and the gross observation of specimen at 4 weeks after surgery. (a) Exposure of the patellar tendon. (b) Preparation of the middle part of patellar tendon. (c) Retraction of the broken end after severing the middle part of the patellar tendon. (d) Sutures after implanting BMSCs. (e–h) Gross observation of the PBS group (e), the normoxia group (f), the hypoxia group (g), and the control (normal) group (h).

The two hind limbs of each rabbit were operated for patellar tendon injury model (*n* = 12 in each group). Rabbits were anesthetized with chloral hydrate (500 mg/kg, i.p.). Chloral hydrate was chosen because the former anesthetic-pentobarbital sodium is a controlled drug and is unavailable to buy now, and our laboratory has no multipurpose device for inhalation anesthesia. A longitudinal skin incision over the patellar tendon was made. Subcutaneous fascia was longitudinally severed (Figure 2(a)). The middle part of the patellar tendon was transversely severed (Figure 2(b)). The retraction of the broken end was 2 mm (Figure 2(c)). 50 *μ*l PBS with hypoxic BMSCs or normoxic BMSCs (1 × 10^6^) was injected into the wound gap followed by suturing the subcutaneous fascia (Figure 2(d)). After the skin was sutured, plaster casts were performed with the knee in full extension. Postoperatively, the rabbits were replaced to their own cages, softly touched every day, and given an intramuscular injection of cefazolin sodium (0.1 g/kg, q.d) for 3 days. The plaster casts were removed after 4 weeks of immobilization.

### 2.10. Histological Analysis

Rabbits were suffocated at 4 weeks after surgery for histological analysis. The surgical site (i.e., middle part) of the patellar tendon was collected, fixed with 4% formalin, dehydrated using graded ethanol, vitrified using dimethylbenzene, and embedded in paraffin. For hematoxylin and eosin (H&E) staining, transverse paraffin sections with a thickness of 4 *μ*m were dewaxed, hydrated, and stained with hematoxylin and eosin. After dehydration and transparency, the sections were sealed with neutral balsam. Histological scores based on previous studies were evaluated according to collagen fiber structure, vascularity, and cellularity of the repaired tendons [25, 26]. The parameters were quantified using a scale ranged from 0 to 3, with 0 being normal pattern, 1 being slightly, 2 being moderately, and 3 being severely fragmented fiber or increased vascularity or increased cellularity. Three sections from each sample spaced at an interval of 50 *μ*m were selected and were evaluated by 3 of the authors (LH, SNW, and WNL) in a blinded manner. For Masson's trichrome staining, the sections were stained according to the manufacturer's protocol (Beijing Solarbio Science & Technology Co., Ltd., China). The collagen fibers and muscle fibers were stained in blue and in red, respectively. For immunohistochemical staining, transverse paraffin sections were incubated in citrate antigen retrieval solution at 95°C for 20 min. Endogenous peroxidase was blocked using 3% hydrogen peroxide for 15 min. After blocking with 10% normal goat serum (BOSTER Biological Technology Co., Ltd., China), the sections were incubated with antibodies to Col-1a1 and Tnmd (Bioss, Beijing, China) overnight at 4°C. After rewarming at room temperature for 30 min, the sections were incubated with goat anti-rabbit horseradish peroxidase-conjugated secondary antibody (BOSTER Biological Technology Co., Ltd., China) for 30 min at room temperature. The diaminobenzidine (Dako, Glostrup, Denmark) working solution was used for color development. The nuclei were stained with hematoxylin. The semiquantitative analysis of immunohistochemical staining was performed with ImagePro Plus version 6.0 (Media Cybernetics, Inc.). At least 3 fields in each sample were analyzed. The average optical density (AOD) in each field was equal to integrated optical density over area.

### 2.11. Biomechanical Testing

Rabbits were suffocated at 4 weeks after surgery for biomechanical analysis as described in our previous study [27]. The patella-patellar tendon-tibial tubercle was harvested and stored at −80°C. The specimens were kept at 4°C overnight prior to the biomechanical test. After measuring the length and width of the patellar tendon, the biomechanical property was tested with the patella and the tibial tubercle mounted on aluminum clamps of a biomechanical testing machine (SPL-10 KN, Shimadzu, Japan). The patellar tendon was loaded along the vertical axis until failure at a displacement rate of 10 cm/min. The maximum load to failure, cross-sectional area, maximum stress, stiffness at failure, and elastic modulus were calculated.

### 2.12. Transmission Electron Microscopy (TEM) Analysis

TEM was used to analyze the diameter of collagen fibrils in regenerated tendons. The sample was first fixed in 2.5% glutaraldehyde solution at 4°C overnight and then was postfixed with 1% OsO4 solution for 1 h. After the sample was dehydrated by a graded series of ethanol, it was infiltrated by acetone and embedding medium and embedded with absolute embedding medium at 37°C overnight and then at 65°C for 48 h. At least 3 sections with a thickness of 60 *μ*m were obtained from each sample using ultramicrotome (Leica Leica UC7, Germany) and were stained by uranyl acetate and alkaline lead citrate for 15 min, respectively, and the cleanest section was selected for TEM test (Hitachi HT7700, Japan). The software ImagePro Plus version 6.0 (Media Cybernetics, Inc.) was used to measure the fiber diameter. After the picture was taken from the TEM, it was converted and calibrated using ImagePro Plus. After selecting measurements and “Automatic Bright Objects”, the number of fibers and the diameter of fibers were calculated automatically.

### 2.13. Statistical Analysis

SPSS version 16.0 was used in this study. Data were identified as homogenous variances and normal distribution when *p* > 0.1 using Levene's test and the Shapiro-Wilk test. All data were presented as the mean ± standard deviation (SD). Independent-sample *t*-test or Mann–Whitney *U*-test was used depending on whether the data followed normal distribution and homogenous variances simultaneously. Linear regression (Pearson's *R*) was used to evaluate the relationship between histological and biomechanical property. *p* < 0.05 was considered to be statistically significant.

## 3. Results

### 3.1. Identification of AMSCs and BMSCs

**Figure 3:**
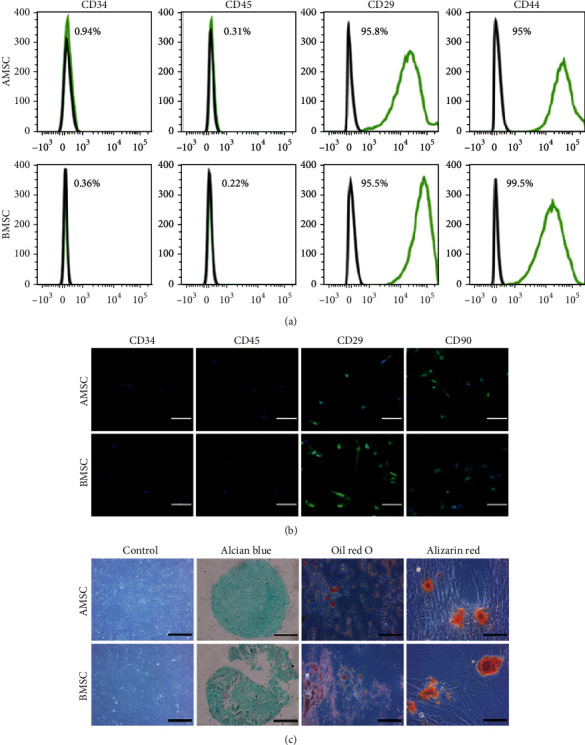
Identification of AMSCs and BMSCs. (a) Flow cytometric analysis of immunophenotypic profiles of AMSCs and BMSCs. (b) Laser scanning confocal microscopic images of immunofluorescence staining for MSC-specific markers CD29 and CD90 and hematopoietic lineage markers CD34 and CD45. Scale bar = 50 *μ*m. Magnification: ×400. (c) Light micrographs and trilineage differentiation of AMSCs and BMSCs to chondrocytes stained with Alcian Blue, adipocytes stained with Oil Red O, and osteocytes stained with Alizarin Red. Control and Alcian Blue staining: scale bar = 200 *μ*m; magnification: ×200. Oil Red O and Alizarin Red staining: scale bar = 100 *μ*m; magnification: ×400.

Analysis of AMSC and BMSC characteristics was performed using flow cytometry and immunofluorescence staining. Flow cytometry analysis showed that both MSCs expressed a series of markers which are considered to be specific to mature MSCs, including CD29 (95.8% in AMSCs, 95.5% in BMSCs) and CD44 (95% in AMSCs, 99.5% in BMSCs) but did not express hematopoietic lineage markers CD34 (0.94% in AMSCs, 0.36% in BMSCs) and CD45 (0.31% in AMSCs, 0.22% in BMSCs) (Figure 3(a)). Laser scanning confocal microscopic images of immunofluorescence-labeled AMSC and BMSC detected positive signals for MSC-specific markers CD29 and CD90 and no signal for CD34 and CD45 (Figure 3(b)). To examine the multipotential differentiation capability, AMSCs and BMSCs were cultured in a commercial induction medium in order to induce differentiation towards chondrogenic, adipogenic, and osteogenic lineages. Both MSCs exhibited fibroblast-like morphology under light microscope. Treated with chondrogenic differentiation media, both AMSCs and BMSCs contained plenty of cartilage-specific acid mucopolysaccharide stained with Alcian Blue solution. Under an adipogenic induction medium, they contained red lipid droplet after Oil Red O staining. Upon osteogenic induction treatment, they were able to mineralize and deposit red calcium nodules, as identified by Alizarin Red staining (Figure 3(c)).

### 3.2. Hypoxia Promoted the Expression of Hif-1*α*

**Figure 4:**
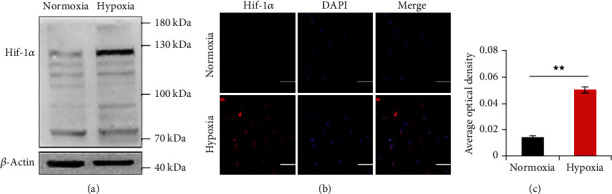
The expression of Hif-1*α* at 7 days after hypoxic induction. Representative western blots (a) and immunofluorescence staining (b) of Hif-1*α* (red) and DAPI-labeled nuclei (blue) and quantification data of immunofluorescence staining (c) under normoxia and hypoxia condition in BMSCs. Scale bar = 50 *μ*m; Magnification: ×400. Data were shown as mean ± SD. ^★^*p* < 0.05; ^★★^*p* < 0.01, vs. the normoxia condition.

To establish hypoxia condition, we examined the expression level of Hif-1*α* by western blot and immunofluorescence staining using laser scanning confocal microscope in BMSCs at 7 days after hypoxic induction. Western blot showed that Hif-1*α* was nearly not expressed in normoxic BMSCs in the vicinity of 120 kDa, whereas it was abundantly expressed in hypoxic BMSCs (Figure 4(a)). Immunofluorescence staining showed the same results to western blot analysis (Figure 4(b)). Quantitative analysis of immunofluorescence staining detected that the AOD of Hif-1*α* was significantly higher under hypoxia condition compared with the normoxia condition (Figure 4(c)).

### 3.3. Hypoxia Increased the Expression Level of Tenogenic Differentiation Markers in Both MSCs In Vitro

Col-1a1, Col-3a1, Dcn, and Tnmd were used as tenocyte-lineage markers. Tenogenic differentiation was assessed based on the expression of these markers at both mRNA and protein levels at 7 days after induction (Supplementary Figure 1-8).

#### 3.3.1. Hypoxia Promoted Tenogenic Differentiation of AMSCs and BMSCs

**Figure 5:**
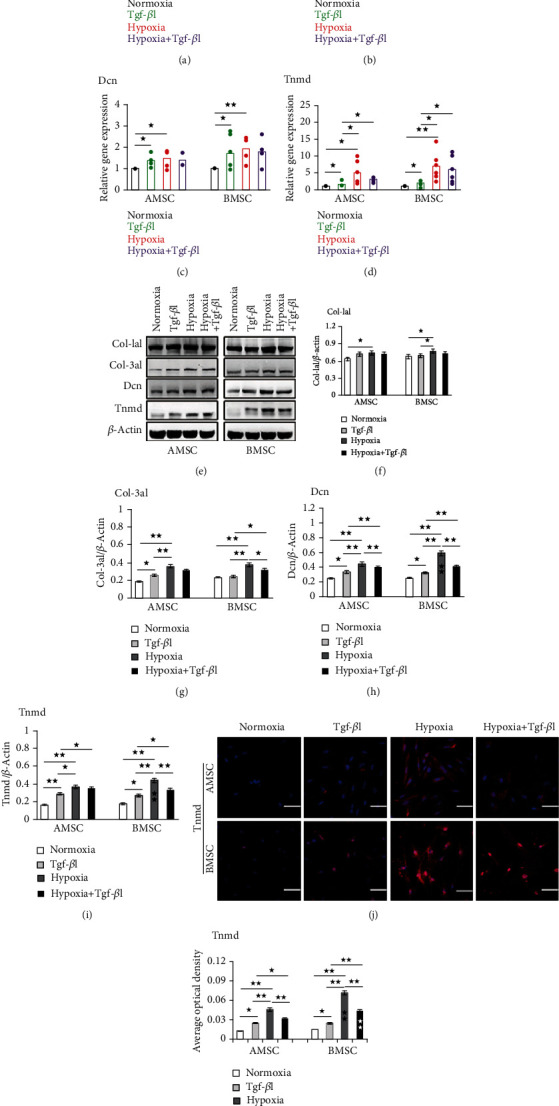
The mRNA and protein expressions of tenogenic markers in AMSCs and BMSCs at 7 days after induction. (a–d) qRT-PCR analysis of gene expression of Col-1a1 (a), Col-3a1 (b), Dcn (c), and Tnmd(d) under normoxia, Tgf-*β*1, hypoxia, and hypoxia+Tgf-*β*1 condition in AMSCs and BMSCs. (e–i) Representative western blots (e) and quantification of protein expression of Col-1a1 (f), Col-3a1 (g), Dcn (h), and Tnmd(i) under normoxia, Tgf-*β*1, hypoxia, and hypoxia+Tgf-*β*1 condition in AMSCs and BMSCs. Asterisks in the column in BMSCs indicated significant difference from the same condition in AMSCs. (j, k) Representative immunofluorescence staining (j) of Tnmd (red) and DAPI-labeled nuclei (blue), and quantification data (k) under under normoxia, Tgf-*β*1, hypoxia, and hypoxia+Tgf-*β*1 condition in AMSCs and BMSCs. Asterisks in the column in BMSCs indicated significant difference from the same condition in AMSCs. Scale bar = 50 *μ*m; Magnification: ×400. Data were shown as mean ± SD. ^★^*p* < 0.05; ^★★^*p* < 0.01.

As shown in Figures 5(a)–5(d), the mRNA levels of all four tenogenic genes in both MSCs were significantly increased in hypoxia induction than those in normoxia. In addition, the mRNA levels of Tnmd in hypoxia induction was the highest among the four genes in both MSCs. Significantly upregulated protein expression levels of all four tenogenic markers in both MSCs were also observed in hypoxia induction than those in normoxia (Figures 5(e)–5(i)). Similar results were found in immunofluorescence staining, which showed that the AOD of Tnmd in hypoxic AMSCs and hypoxic BMSCs was significantly higher than that in normoxic AMSCs and normoxic BMSCs, respectively (Figures 5(j) and 5(k)).

#### 3.3.2. Hypoxic BMSCs Exhibited the Higher Potential of Tenogenic Differentiation than Hypoxic AMSCs

As shown in Figures 5(a)–5(d), the increased mRNA levels of all four tenogenic genes in hypoxic BMSCs were higher than those in hypoxic AMSCs, although the differences were not remarkable. However, the protein levels of Dcn and Tnmd were significantly higher in hypoxic BMSCs than those in hypoxic AMSCs (Figures 5(h) and 5(i)). The protein level of Tnmd examined by western blot was similar to that detected by laser scanning confocal microscope, under which the AOD of Tnmd in hypoxic BMSCs was significantly higher than that in hypoxic AMSCs (Figures 5(j) and 5(k)).

#### 3.3.3. Hypoxia Showed the Higher Inductility Compared with Tgf-*β*1

As shown in Figures 5(a)–5(d), the mRNA levels of all four tenogenic genes in both MSCs were higher in hypoxia induction than those in Tgf-*β*1 induction, although only the difference of Tnmd was significant. However, western blot analysis showed that the expression of protein level of the four tenocyte-lineage markers in both MSCs were significantly higher in hypoxic induction than those in Tgf-*β*1 induction (Figures 5(g)–5(i)), except Col-1a1 in AMSCs (Figure 5(f)). Immunofluorescence staining detected that the AOD of Tnmd in both MSCs was significantly higher in hypoxia induction than in Tgf-*β*1 induction (Figures 5(j) and 5(k)), which was similar to the western blot analysis.

#### 3.3.4. Hypoxia and Tgf-*β*1 Did Not Induce Synergistically MSCs to Tenogenic Differentiation

Tgf-*β*1 enhanced the mRNA expression of Col-1a1 in BMSCs (Figure 5(a)), the protein expression of Col-3a1 in AMSCs (Figure 5(g)), and the mRNA and protein expression of Dcn and Tnmd in AMSCs and BMSCs (Figures 5(c), 5(d), 5(h), and 5(i)). Hypoxia promoted the ability of Tgf-*β*1 to induce tenogenic differentiation, as shown by the mRNA and protein levels of all four tenogenic genes, and the AOD of Tnmd in both MSCs was higher in hypoxia and Tgf-*β*1 induction than in Tgf-*β*1 induction alone (Figures 5(a)–5(k)), except for the mRNA expression of Col-3a1 in AMSCs (Figure 5(b)). However, Tgf-*β*1 inhibited the ability of hypoxia to induce tenogenic differentiation, as shown by the mRNA and protein levels of all four tenogenic genes and the AOD of Tnmd in both MSCs were lower in hypoxia and Tgf-*β*1 induction than those in hypoxia induction alone (Figures 5(a)–5(k)).

### 3.4. Hypoxia-Induced BMSCs Promoted Patellar Tendon Repair

The effect of hypoxic BMSCs on tenogenesis was examined in vivo. Normoxic and hypoxic BMSCs were cultured for 7 days and then were injected into the wound gap of patellar tendon after surgery (Figures 2(a)–2(d)), respectively. After 4 weeks, the repaired tendons with patella and tibial tubercle were removed for further analysis.

#### 3.4.1. Macroscopic Inspection and H&E Staining

**Figure 6:**
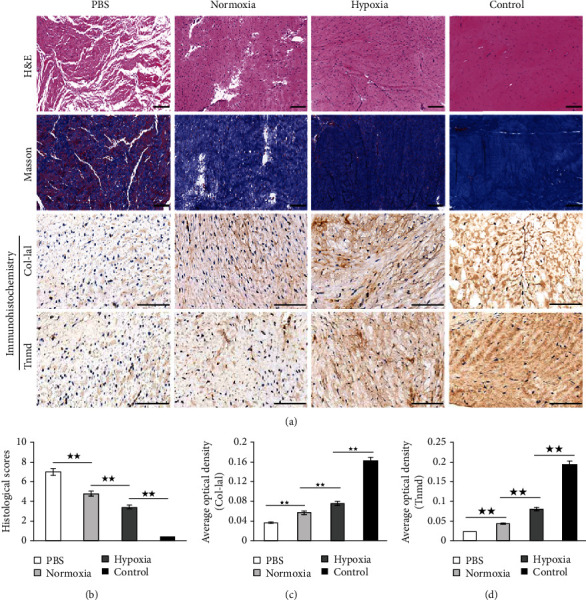
Histological analysis of specimen at 4 weeks after surgery. (a) Hematoxylin and eosin (H&E) staining, Masson's trichrome staining, and immunohistochemical staining for Col-1a1 and Tnmd in the PBS group, the normoxia group, the hypoxia group, and the control (normal) group. (b) Quantification of histological properties after H&E staining in the PBS group, the normoxia group, the hypoxia group, and the control (normal) group. (c, d) Quantification of Col-1a1 (c) and Tnmd (d) after immunohistochemical staining in the PBS group, the normoxia group, the hypoxia group, and the control (normal) group. H&E and Masson's trichrome staining: scale bar = 200 *μ*m; magnification: ×200. Immunohistochemical staining: scale bar = 200 *μ*m; magnification: ×400. Data were shown as mean ± SD. ^★★^*p* < 0.01.

As shown in Figures 2(e)–2(h), there was a remarkable defect in the PBS group at 4 weeks after surgery. However, when applied normoxic BMSCs into the wound gap, the injured tendon was repaired better than that in the PBS group under gross observation. The repair was further improved in the hypoxia group, which was still obviously different with the normal tendon. The difference between the four groups in tendon repair was evaluated by H&E staining. As shown in Figure 6(a), there were large empty spaces and relatively fewer cells within irregularly arranged collagen fibers in the PBS group. In addition, bold vessels extended into empty spaces were observed. In the normoxia group, the collagen fiber became compact, and fewer empty spaces and more cells were found. Hypercellularity of repaired tendon remained present in the hypoxia group. The arrangement of collagen fiber was further improved, as shown by few vessels and empty spaces. However, the histological properties of the hypoxia group were still remarkably different to those in the control group, which had regularly arranged fibers and fewer cells and vessels. Quantitative analysis showed that the histological scores of the PBS group, the normoxia group, the hypoxia group and the control group were 7 ± 0, 4.8 ± 0.45, 3.4 ± 0.55, and 0.4 ± 0.55, respectively. The histological score of the normoxia group was significantly lower than in the PBS group but was significantly higher than in the hypoxia group. The histological score of the hypoxia group was significantly higher than in the control group (Figure 6(b)).

#### 3.4.2. Masson's Trichrome Staining

Masson's trichrome staining was used in order to evaluate the formation of tendon-like tissues. As shown in Figure 6(a), few formation of collagen (shown in blue) was seen in the PBS group. By contrast, a large amount of muscle fibers (shown in red) was observed instead. In the normoxia group, more tendon-like tissues were deposited but were apparently fewer than in the hypoxia group. In the control group, the patellar tendon was abundant in the fibrous matrix which was stained in blue, whereas very few muscle fibers occurred in the tendon.

#### 3.4.3. Immunohistochemical Staining

As shown in Figure 6(a), immunohistochemical staining of Col-1a1 and Tnmd was applied to examine the difference of tenogenic differentiation in the four groups. Both Col-1a1 and Tnmd were slightly stained in the PBS group. The staining of Col-1a1 and Tnmd was deeper in the normoxia group and became more deep in the hypoxia group. Quantitative analysis of the average optical density (AOD) of Col-1a1 (Figure 6(c)) and Tnmd (Figure 6(d)) found that the PBS group showed the lowest AOD among the four groups. The AOD of the hypoxia group was significantly higher than the nomoxia group. The staining of Col-1a1 and Tnmd was the deepest in the control group, which had the highest AOD in the four groups.

#### 3.4.4. Ultrastructural Morphology of Collagen Fibrils

**Figure 7:**
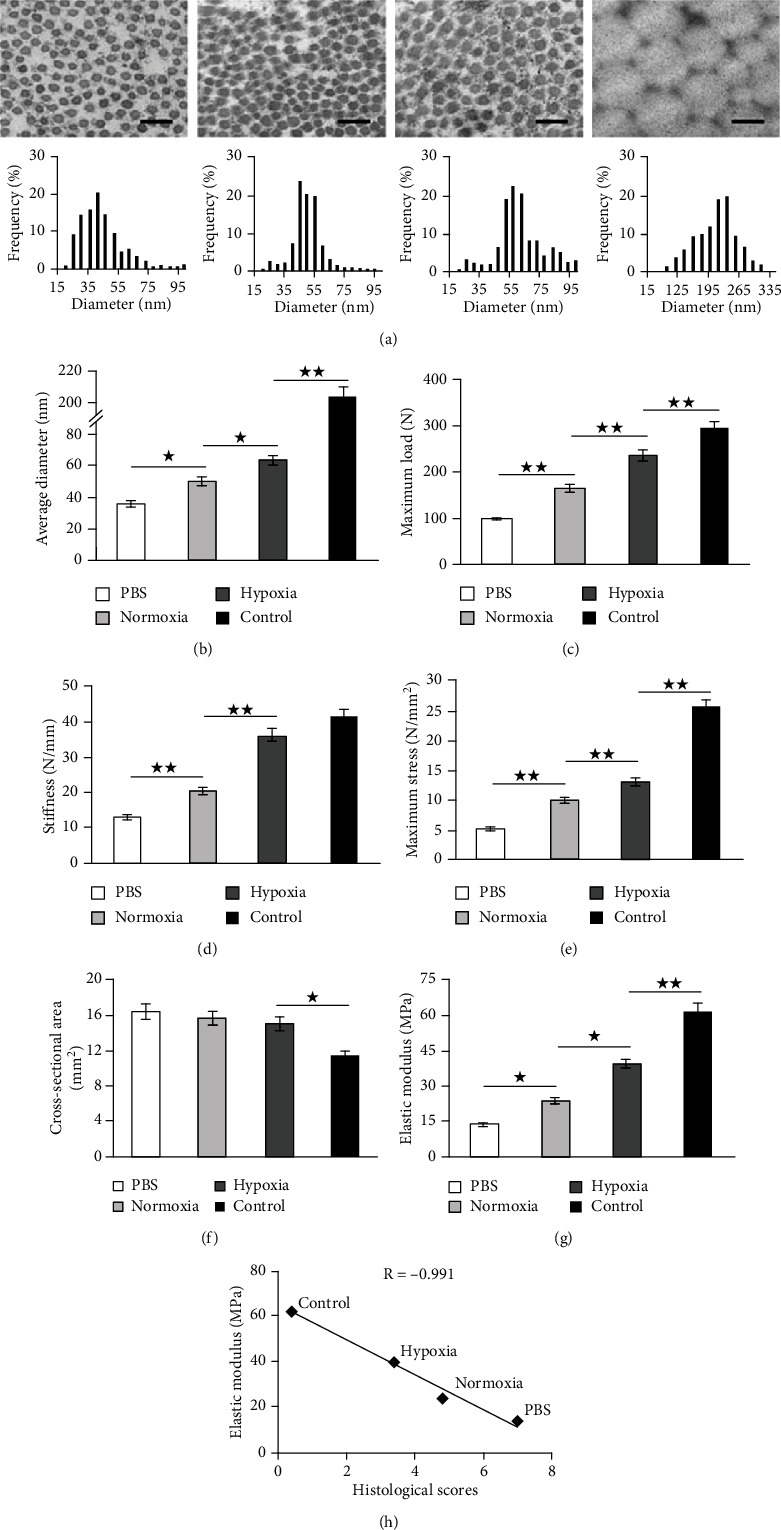
Ultrastructure and biomechanical analysis of specimen at 4 weeks after surgery. Representative images of transmission electron microscopy and the distribution of collagen fibril diameters (a) and the average diameter of collagen fibrils (b) in the PBS group, the normoxia group, the hypoxia group, and the control (normal) group. Scale bar = 200 nm. Magnification: ×15000. (c–g) Biomechanical analysis for maximum load to failure (c), stiffness at failure (d), maximum stress (e), cross-sectional area (f), and elastic modulus (g) in the PBS group, the normoxia group, the hypoxia group, and the control (normal) group. (h) Regression analysis of the relationship between histological scores and elastic modulus. The points represented the mean of each group. Data were shown as mean ± SD.^★^*p* < 0.05; ^★★^*p* < 0.01.

The diameters of collagen fibrils in repaired tendon determined the biomechanical properties of the tendon. As a result, transmission electron microscopy (TEM) was used to analyze the diameters of the fibrils at 4 weeks after surgery. We calculated the range of collagen fibril diameters and found that most diameters of collagen fibrils ranged from 25 to 46 nm in the PBS group, 45 to 55 nm in the normoxia group, 53 to 73 nm in the hypoxia group, and 159 to 256 nm in the control group (Figure 7(a)). The average diameter of collagen fibrils in the normoxia group was significantly larger than that in the PBS group but was significantly smaller than that in the hypoxia group. However, the average diameter of collagen fibrils in the hypoxia group was remarkably smaller than that in the control group (Figure 7(b)).

#### 3.4.5. Biomechanical Properties of Repaired Tendons

The maximum load to failure (Figure 7(c)), stiffness at failure (Figure 7(d)), maximum stress (Figure 7(e)), and elastic modulus (Figure 7(g)) were significantly higher in the normoxia group, compared with those in the PBS group but were significantly lower in the hypoxia group, which remained significantly lower in the control group except for stiffness. However, the cross-sectional area (Figure 7(f)) was significantly larger after surgery, with the largest in the PBS group, the smallest in the hypoxia group which was still significantly larger than that in the control group. The maximum load to failure, stiffness at failure, maximum stress, and elastic modulus in the PBS group at 4 weeks after surgery exhibited 32.8%, 31.5%, 20.3%, and 22.3%, respectively, of those in the control group, whereas those in the normoxia group exhibited 56%, 49.3%, 38.9%, and 38.45%, respectively. The biomechanical properties were improved persistently in the hypoxia group, evidenced by the finding that the maximum load to failure, stiffness at failure, maximum stress, and elastic modulus exhibited 80.2%, 87.5%, 50.9%, and 63.5%, respectively, of those in the control group. In addition, the correlation between histological scores and elastic modulus was evaluated. As shown in Figure 7(h), the results from each group demonstrated that there was a strong and negative linear correlation between the decreasing scores and the increasing elastic modulus.

## 4. Discussion

Although different findings have been reported in the trilineage differentiation of MSCs in hypoxia induction [17, 20], few, if any, studies have conducted the tenogenic differentiation of MSCs and the different potential of tenogenic differentiation of different MSCs in hypoxia condition. In this study, we showed that the associated markers of tenogenesis, such as Col-1a1, Col-3a1, Dcn, and Tnmd, were significantly increased in hypoxic AMSCs and hypoxic BMSCs in vitro, compared with those in normoxic MSCs and that hypoxic BMSCs exhibited better histological and biomechanical properties than normoxic BMSCs in vivo. Our in vitro data further demonstrated that the inductivity of hypoxia in tenogenic differentiation of AMSCs and BMSCs was significantly greater than that of Tgf-*β*1 and that hypoxic BMSCs had greater potential of tenogenic differentiation than hypoxic AMSCs.

Few specific markers have been identified in tenogenesis. Col-1a1 and Col-3a1 are considered to be the main tendon collagen in matrix [5], especially Col-1a1 which has often been used as a tendon-associated marker in tenogenic differentiation study [14, 28]. Tnmd is a member of type II transmembrane glycoprotein. The reduced cell numbers and the altered structure of collagen fibrils in adult tendons of Tnmd-deficient mice suggested Tnmd was a marker of differentiated tenocytes in mature tendon [29]. Dcn is the most abundant proteoglycan in the tendon tissue. The fact that decorin-deficient mice showed aberrant collagen fibrils indicated that decorin binding was required for appropriate assembly of collagen fibrils [30]. In our study, we analyzed the expression characteristic of those tenogenic markers in hypoxia induction and detected that the mRNA and protein levels of Col-1a1, Col-3a1, Dcn, and Tnmd were significantly increased in hypoxia treatment than those in normoxia condition. Our data which resulted from the separate effect of hypoxia were consistent with a previous study which found that, in an indirect coculture with tenocytes, hypoxia significantly increased the gene expression levels of Col-1a1, Col-3a1, and Tnmd in human AMSCs [31]. Our results also demonstrated that Tnmd had the highest mRNA expression, which showed nearly 5-fold to control in AMSCs and more than 5-fold to control in BMSCs, whereas Col-1a1, Col-3a1, and Dcn all exhibited less than 2-fold to control. Our findings were similar to a previous study which found that compared with E11.5 tendon progenitor cells, Tnmd was the second most differentially expressed gene, displaying a 376-fold change in the top 100 upregulated genes in E14.5 differentiated tendon cells, whereas Dcn and Col-1a1 displayed a 36-fold change and a 14-fold change, respectively [32]. Considering the also significantly increased protein levels of Tnmd in hypoxia induction shown in western blot analysis and immunohistochemical staining, we believed hypoxia promoted tenogenic differentiation of MSCs mainly by enhancing the expression of Tnmd. This paralleled the finding that human tendon-derived stem cells expressed a significantly higher level of Tnmd, instead of tenascin-C and scleraxis, in hypoxia induction than in normoxia induction [21].

The biological characteristic of AMSC and BMSC has been reported to be significantly different. The concentration of AMSCs in adipose tissue is about 2%, while BMSCs constitute very little proportion of the cells, about 0.01–0.001% in the bone marrow [33, 34]. AMSCs possessed the higher proliferation potential and colony frequency [33, 35] and exhibited greater tolerance to apoptosis [36], compared with BMSCs. The fact that regenerated tendon treated with BMSCs exhibited an earlier improvement and a higher mRNA expression of Col-1a1and Dcn compared with AMSCs indicated that the potential of tenogenic differentiation of BMSCs was significantly stronger than that of AMSCs [22]. Our results demonstrated the similar result that the potential of tenogenic differentiation of hypoxic BMSCs was also significantly stronger than that of hypoxic AMSCs. The finding suggested that BMSCs were the optimal cell sources for improving tendon repair via hypoxia induction, instead of AMSCs. However, our results were inconsistent with another report which found that the fluorescence intensity of Dcn in AMSCs was significantly increased compared with that in BMSCs after 3 days of tenogenic induction [37]. This indicated that further research is needed to determine the best source of MSCs used for inducing tenogenic differentiation.

In order to better understand the role of hypoxia in tenogenesis, we compared the inductivity of tenogenic differentiation between hypoxia and Tgf-*β*1 and revealed that the protein levels of Col-1a1 in BMSCs and Col-3a1, Dcn, and Tnmd in both AMSCs and BMSCs under hypoxia condition were all significantly higher than those in the presence of Tgf-*β*1. A previous study has found that the mRNA expression levels of Tnmd in BMSCs at 7 days did not show any significant difference with that at 3 days after Tgf-*β*1 induction [24]. This result suggested that the potential of tenogenic differentiation of MSC did not increase with induction time of Tgf-*β*1. Recent data showed that after Tgf-*β*1 administrations in a rat rotator cuff repair model, the mRNA levels of Col-1a1, Col-3a1, and Tnmd in tendon formation were slightly increased at all timepoints after surgery, but with no significant difference compared with the normal tendon. However, the expression of matrix metalloproteinase-9 (MMP-9) and MMP-13 was significantly decreased at 2 weeks postoperatively [38]. These findings indicated that Tgf-*β*1 increased collagen accumulation by inhibiting MMP-9 and MMP-13 expressions to enhance tendon formation at the healing site. These may be possible explanations why Tgf-*β*1 had a relative low inductivity in comparison to hypoxia which directly promoted the expression of tenogenic markers of MSCs and thus resulted in better recovery of injured tendon in our study. In addition, our results showed that the combination of hypoxia and Tgf-*β*1 induction for 7 days demonstrated a greater increase than Tgf-*β*1 induction, but a smaller increase than hypoxia induction in gene expression of teongenic differentiation markers in both AMSCs and BMSCs. This was partly similar to another study which found that low oxygen culture with Tgf-*β*3 addition (10 ng/ml) for a period of 14 days produced a greater increase in type I and III collagen relative to low oxygen culture and Tgf-*β*3 induction individually in human BMSCs-incorporated fibrin gels [39]. Apart from the difference in the period of cell culture, the fact that Tgf-*β*3 mainly induced the formation of mature tendon composed primarily of type I collagen in tendon healing in comparison with Tgf-*β*1 may also be another reason for the different findings of tenogenic differentiation in hypoxia condition combined with the addition of growth factors to culture media [39, 40].

The higher expression of Col-1a1 in the hypoxic group in vivo in our study was consistent with a previous finding which showed that transplantation of hypoxic BMSCs may be a more effective treatment than normoxic BMSCs for Achilles tendon ruptures in rats [41]. However, our study further demonstrated that hypoxic BMSCs showed a greater ability to differentiate into tendon tissues and improved tendon repair by increasing the production of Tnmd, a marker of mature tendon. In addition, we analyzed the correlation between histological scores and elastic modulus. The *R* value of -0.991 indicated a strong and negative correlation between them. Our results were similar to previous findings that histological scores had a strong and negative linear correlation with ultimate tensile load in healing Achilles tendon rupture in rats [42].

There are several limitations in our study. Tgf-*β*1 was often combined with other inducers for tendon differentiation [14, 43], although its use alone had definite capability to induce tenogenic differentiation which was used to compare with the inductivity of hypoxia in this study. In addition, we did not seed fluorescent-labeled MSCs in the wound gap to investigate the role of hypoxia-induced MSCs in tenogenesis in vivo. However, previous studies reported that BrdU-labeled MSCs [41] or GFP-labeled MSCs [19] can be observed at 4 weeks after transplantation. This indicated that transplanted MSCs can be able to survive in vivo at least at 4 weeks postoperatively and thus contributed to a corresponding role in tendon healing. Therefore, we believe that the histological and biomechanical analyses in the present study, which also lasted 4 weeks after surgery, were the results that came from the role of the transplanted MSCs. Lastly, we only investigated the effects of hypoxia on tendon matrix-associated markers in vitro. No transcription factor and its role in tenogenic differentiation were studied. Further studies are needed to investigate the mechanisms underlying tenogenic differentiation of MSCs in hypoxia induction.

## 5. Conclusion

In conclusion, we have investigated the role of hypoxia in tenogenic differentiation of AMSCs and BMSCs and conducted histological and biomechanical analyses in the patellar tendon injury model. Our data showed that hypoxia can significantly enhance the expression of Col-1a1, Col-3a1, Dcn, and Tnmd in AMSCs and BMSCs in vitro. Hypoxic BMSCs possessed higher levels of tenogenic markers than hypoxic AMSCs. In addition, the expression levels of tenogenic markers in both MSCs in hypoxia were higher than those in Tgf-*β*1. In vivo, hypoxic BMSCs exhibited improved histological appearances and biomechanical properties and thus produced superior healing compared to normoxic BMSCs.

## Figures and Tables

**Table 1:** Primers used in qRT-PCR.

Gene	Primer sequences	Accession no.	Product length (bp)
Col-1a1	F:TGGCGAGCCTGGAGCTTCTG	NC_013687.1	81
	R:GCTTCTCCGTCATCTCCGTTCTTG		
Col-3a1	F:TCCTGGTGCTATTGGTCCGTCTG	NC_013675.1	160
	R:TCCGTCGAAGCCTCTGTGTCC		
Dcn	F:CAGTGTCACCTTCGAGTTGTCCAG	NC_013672.1	89
	R:AGGTCCAGTAGCGTCGTGTCAG		
Tnmd	F: CGCCAGACAAGCAAGTGAGGAAG	NC_013690.1	134
	R: CACGGCGGCAGTAGCGATTG		
*β*-Actin	F: CATCCTGGCCTCGCTCTCCAC	NW_003159504.1	180
	R: AAAGCCATGCCAATCTCGTCT		

F: forward; R: reverse.

## Data Availability

The datasets generated during the current study are available from the corresponding author on reasonable request.

## Supplementary Materials

Supplementary Materials Supplementary Figure 1: Col-1a1 of AMSCs by western blot. Supplementary Figure 2: Col-3a1 of AMSCs by western blot. Supplementary Figure 3: Dcn of AMSCs by western blot. Supplementary Figure 4: *β*-Actin and Tnmd of AMSCs by western blot. Supplementary Figure 5: Col-1a1 of BMSCs by Western blot. Supplementary Figure 6: Col-3a1 of BMSCs by western blot. Supplementary Figure 7: Dcn of BMSCs by western blot. Supplementary Figure 8: *β*-Actin and Tnmd of BMSCs by western blot.
