# Supplementary figures and images for: Hypoxia-Induced Mesenchymal Stem Cells Exhibit Stronger Tenogenic Differentiation Capacities and Promote Patellar Tendon Repair in Rabbits
Source: Stem Cells Int. 2020 Oct 17;2020:8822609. doi: 10.1155/2020/8822609 (PMC7591963; doi:10.1155/2020/8822609)

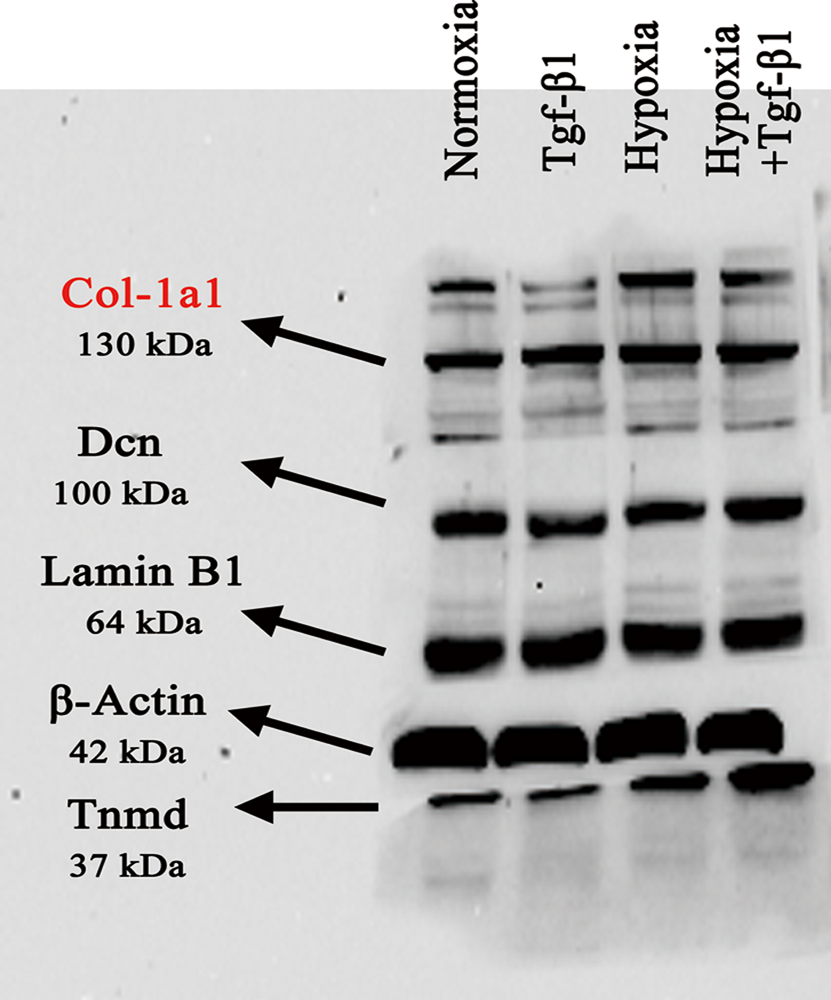

Supplement: Supplementary Materials — Supplementary Figure 1: Col-1a1 of AMSCs by western blot. Supplementary Figure 2: Col-3a1 of AMSCs by western blot. Supplementary Figure 3: Dcn of AMSCs by western blot. Supplementary Figure 4: β-Actin and Tnmd of AMSCs by western blot. Supplementary Figure 5: Col-1a1 of BMSCs by Western blot. Supplementary Figure 6: Col-3a1 of BMSCs by western blot. Supplementary Figure 7: Dcn of BMSCs by western blot. Supplementary Figure 8: β-Actin and Tnmd of BMSCs by western blot. [file 8822609.f1.zip › Supplementary Materials/Supplementary Figure 1/Col-1a1 of AMSCs.tif]

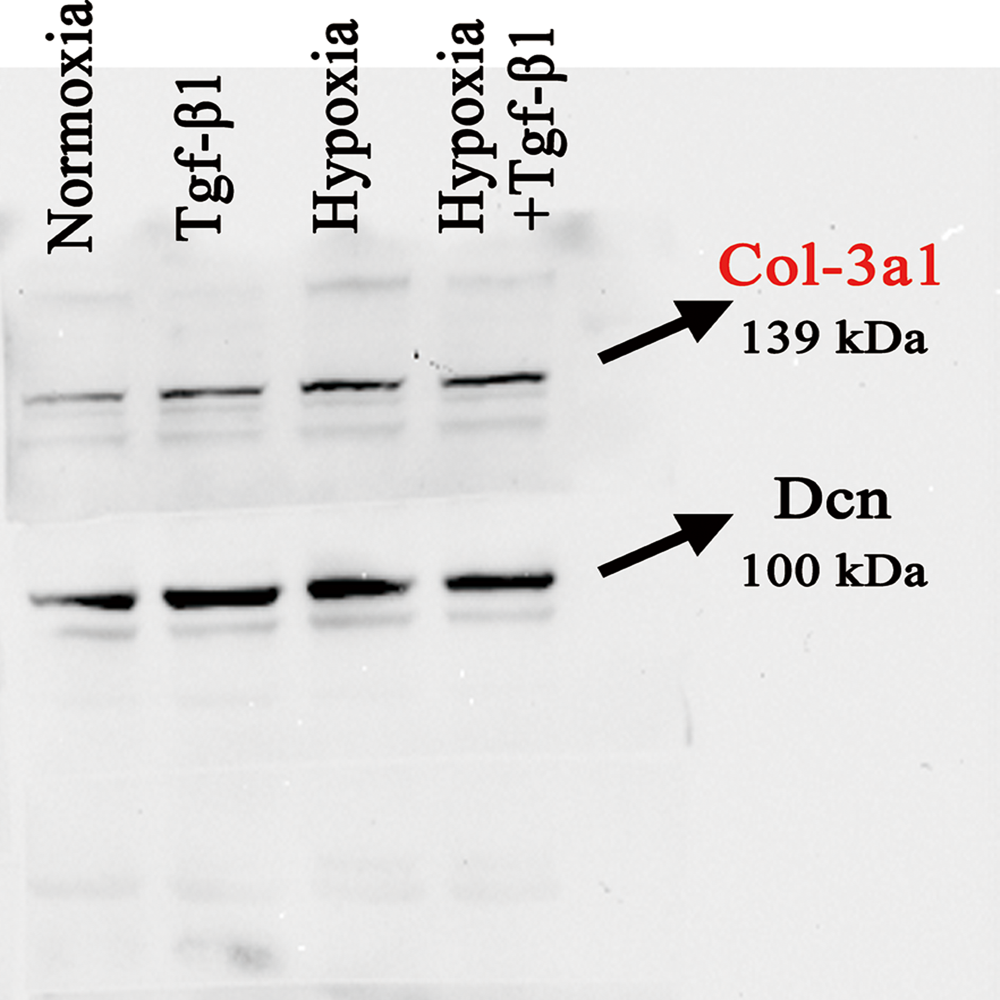

Supplement: Supplementary Materials — Supplementary Figure 1: Col-1a1 of AMSCs by western blot. Supplementary Figure 2: Col-3a1 of AMSCs by western blot. Supplementary Figure 3: Dcn of AMSCs by western blot. Supplementary Figure 4: β-Actin and Tnmd of AMSCs by western blot. Supplementary Figure 5: Col-1a1 of BMSCs by Western blot. Supplementary Figure 6: Col-3a1 of BMSCs by western blot. Supplementary Figure 7: Dcn of BMSCs by western blot. Supplementary Figure 8: β-Actin and Tnmd of BMSCs by western blot. [file 8822609.f1.zip › Supplementary Materials/Supplementary Figure 2/Col-3a1 of AMSCs .tif]

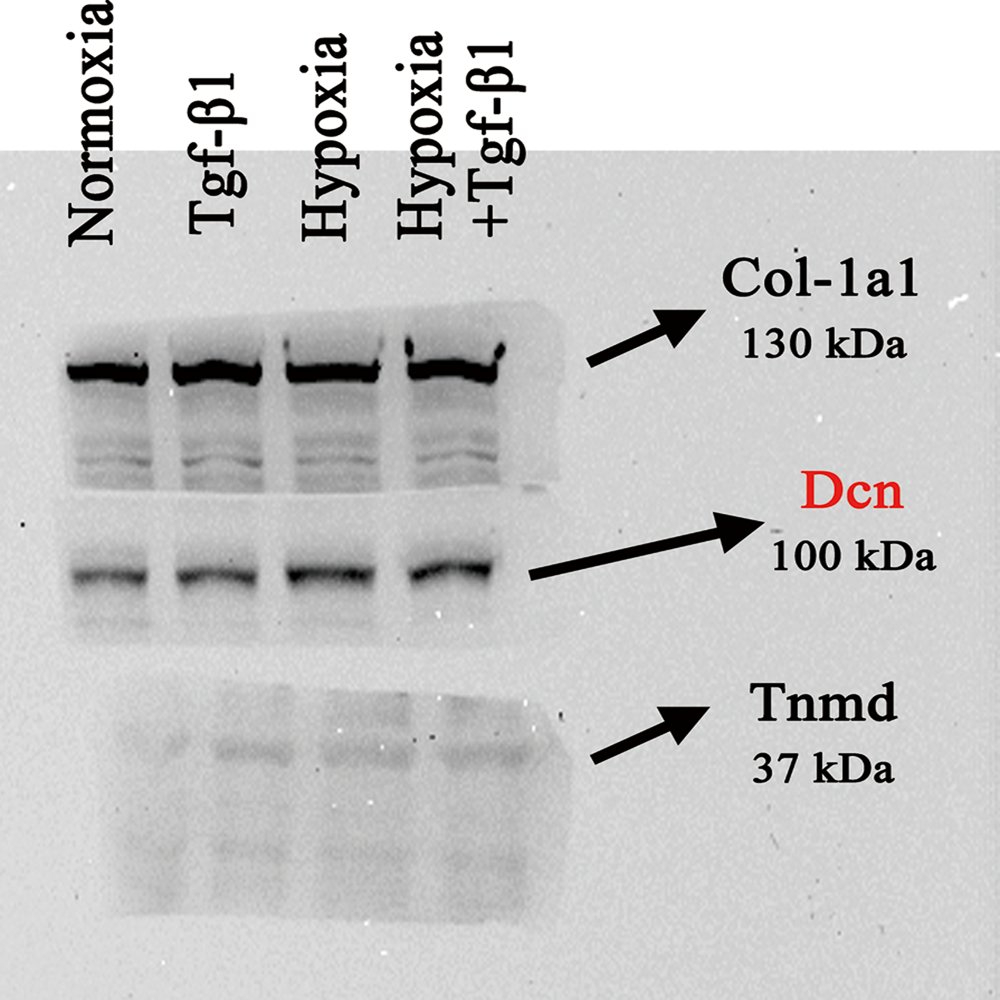

Supplement: Supplementary Materials — Supplementary Figure 1: Col-1a1 of AMSCs by western blot. Supplementary Figure 2: Col-3a1 of AMSCs by western blot. Supplementary Figure 3: Dcn of AMSCs by western blot. Supplementary Figure 4: β-Actin and Tnmd of AMSCs by western blot. Supplementary Figure 5: Col-1a1 of BMSCs by Western blot. Supplementary Figure 6: Col-3a1 of BMSCs by western blot. Supplementary Figure 7: Dcn of BMSCs by western blot. Supplementary Figure 8: β-Actin and Tnmd of BMSCs by western blot. [file 8822609.f1.zip › Supplementary Materials/Supplementary Figure 3/Dcn of AMSCs.tif]

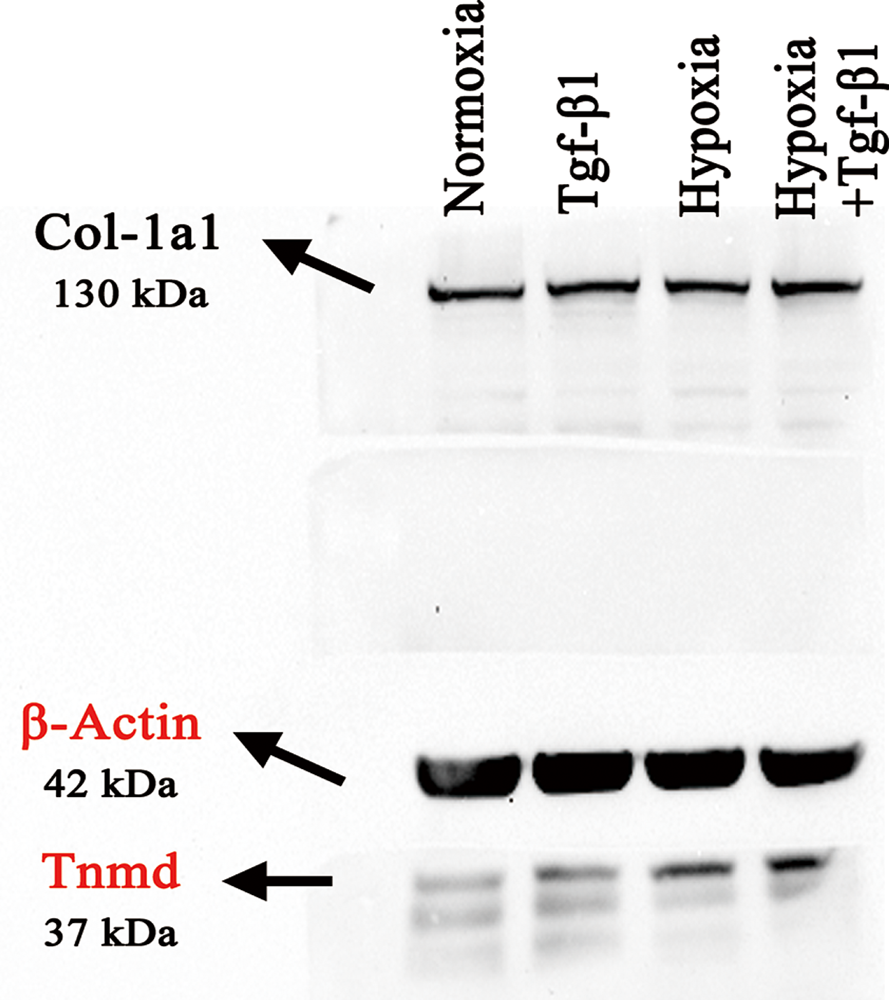

Supplement: Supplementary Materials — Supplementary Figure 1: Col-1a1 of AMSCs by western blot. Supplementary Figure 2: Col-3a1 of AMSCs by western blot. Supplementary Figure 3: Dcn of AMSCs by western blot. Supplementary Figure 4: β-Actin and Tnmd of AMSCs by western blot. Supplementary Figure 5: Col-1a1 of BMSCs by Western blot. Supplementary Figure 6: Col-3a1 of BMSCs by western blot. Supplementary Figure 7: Dcn of BMSCs by western blot. Supplementary Figure 8: β-Actin and Tnmd of BMSCs by western blot. [file 8822609.f1.zip › Supplementary Materials/Supplementary Figure 4/a┬-Actin and Tnmd of AMSCs.tif]

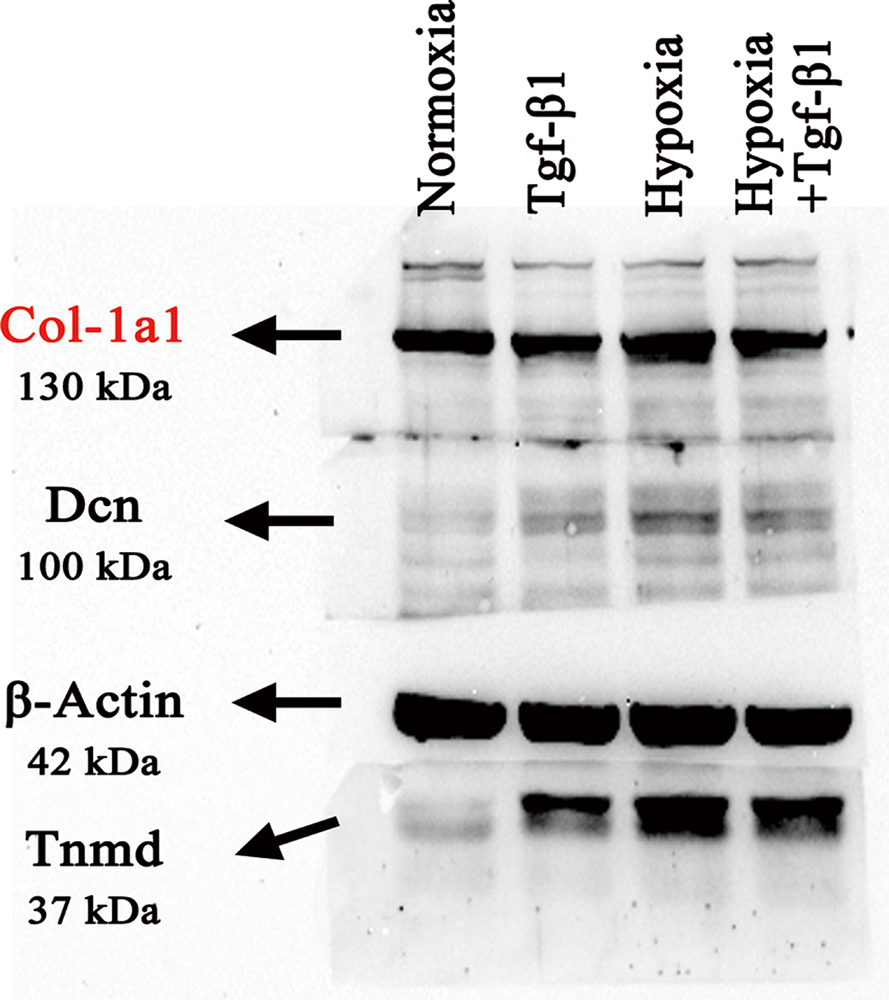

Supplement: Supplementary Materials — Supplementary Figure 1: Col-1a1 of AMSCs by western blot. Supplementary Figure 2: Col-3a1 of AMSCs by western blot. Supplementary Figure 3: Dcn of AMSCs by western blot. Supplementary Figure 4: β-Actin and Tnmd of AMSCs by western blot. Supplementary Figure 5: Col-1a1 of BMSCs by Western blot. Supplementary Figure 6: Col-3a1 of BMSCs by western blot. Supplementary Figure 7: Dcn of BMSCs by western blot. Supplementary Figure 8: β-Actin and Tnmd of BMSCs by western blot. [file 8822609.f1.zip › Supplementary Materials/Supplementary Figure 5/Col-1a1 of BMSCs.tif]

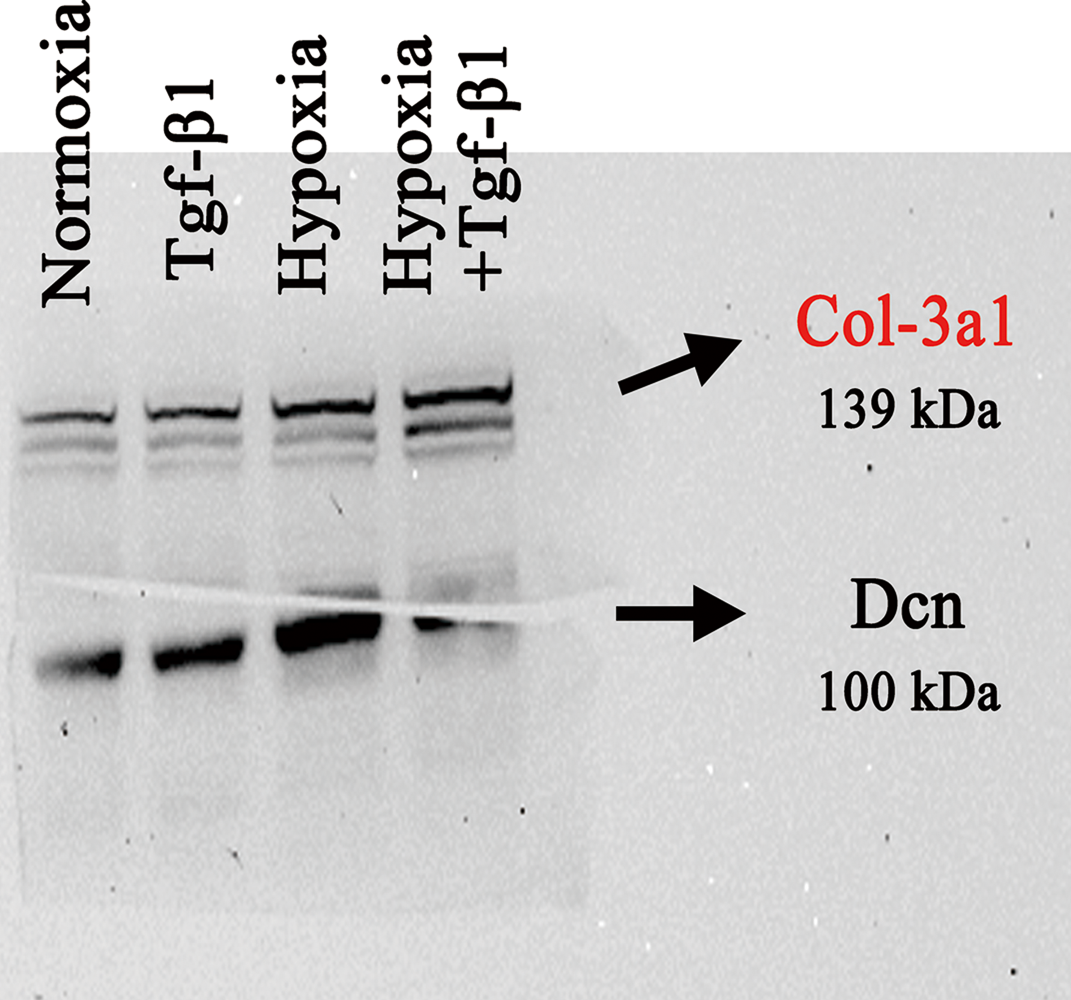

Supplement: Supplementary Materials — Supplementary Figure 1: Col-1a1 of AMSCs by western blot. Supplementary Figure 2: Col-3a1 of AMSCs by western blot. Supplementary Figure 3: Dcn of AMSCs by western blot. Supplementary Figure 4: β-Actin and Tnmd of AMSCs by western blot. Supplementary Figure 5: Col-1a1 of BMSCs by Western blot. Supplementary Figure 6: Col-3a1 of BMSCs by western blot. Supplementary Figure 7: Dcn of BMSCs by western blot. Supplementary Figure 8: β-Actin and Tnmd of BMSCs by western blot. [file 8822609.f1.zip › Supplementary Materials/Supplementary Figure 6/Col-3a1 of BMSCs .tif]

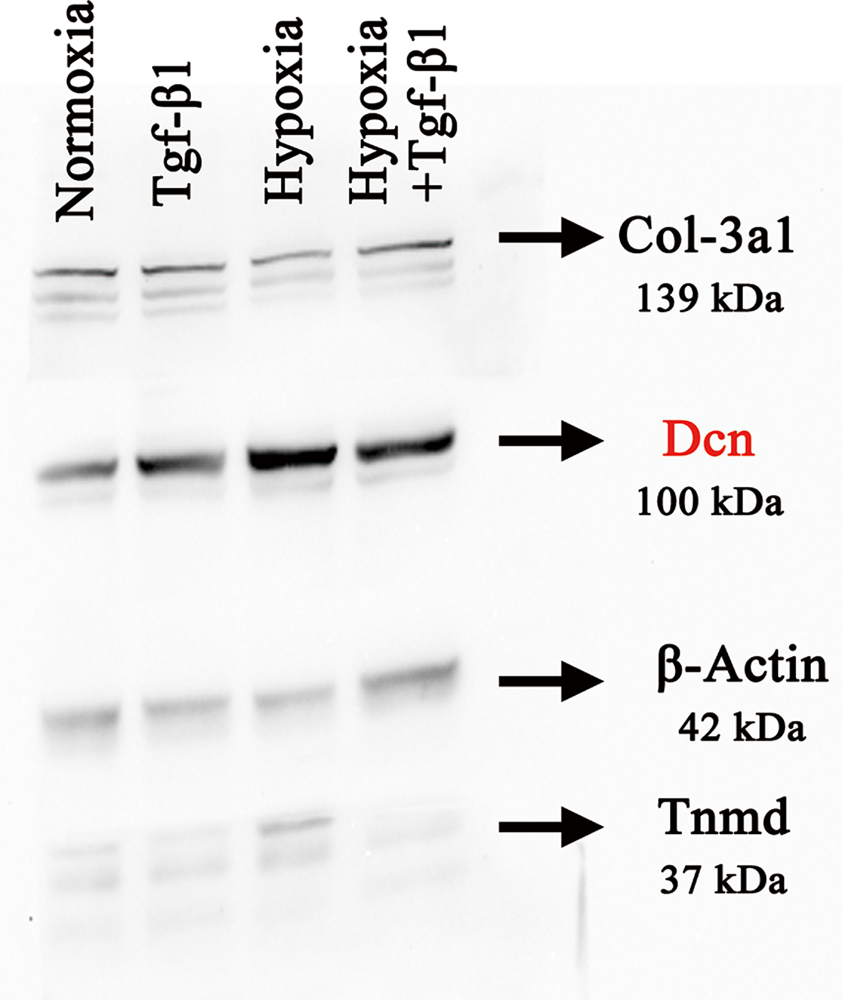

Supplement: Supplementary Materials — Supplementary Figure 1: Col-1a1 of AMSCs by western blot. Supplementary Figure 2: Col-3a1 of AMSCs by western blot. Supplementary Figure 3: Dcn of AMSCs by western blot. Supplementary Figure 4: β-Actin and Tnmd of AMSCs by western blot. Supplementary Figure 5: Col-1a1 of BMSCs by Western blot. Supplementary Figure 6: Col-3a1 of BMSCs by western blot. Supplementary Figure 7: Dcn of BMSCs by western blot. Supplementary Figure 8: β-Actin and Tnmd of BMSCs by western blot. [file 8822609.f1.zip › Supplementary Materials/Supplementary Figure 7/Dcn of BMSCs.tif]

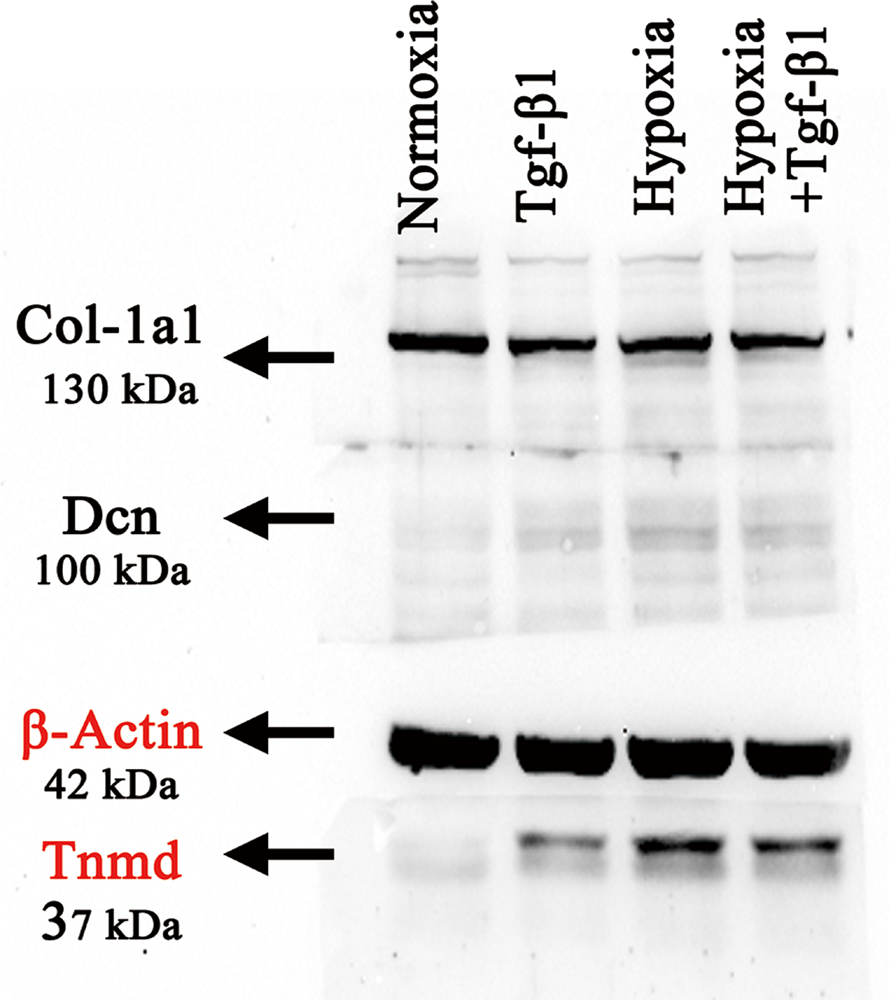

Supplement: Supplementary Materials — Supplementary Figure 1: Col-1a1 of AMSCs by western blot. Supplementary Figure 2: Col-3a1 of AMSCs by western blot. Supplementary Figure 3: Dcn of AMSCs by western blot. Supplementary Figure 4: β-Actin and Tnmd of AMSCs by western blot. Supplementary Figure 5: Col-1a1 of BMSCs by Western blot. Supplementary Figure 6: Col-3a1 of BMSCs by western blot. Supplementary Figure 7: Dcn of BMSCs by western blot. Supplementary Figure 8: β-Actin and Tnmd of BMSCs by western blot. [file 8822609.f1.zip › Supplementary Materials/Supplementary Figure 8/a┬-Actin and Tnmd of BMSCs.tif]
